# Comparison of a qualitative measurement of heart-type fatty acid-binding protein with other cardiac markers as an early diagnostic marker in the diagnosis of non-ST - segment elevation myocardial infarction

**DOI:** 10.5830/CVJA-2015-028

**Published:** 2015

**Authors:** Demet Menekşe Gerede, Sadi Güleç, Mustafa Kılıçkap, Cansın Tulunay Kaya, Veysel Kutay Vurgun, Özgür Ulaş Özcan, Hüseyin Göksülük, Çetin Erol

**Affiliations:** Department of Cardiology, Ankara University School of Medicine, Ankara, Turkey; Department of Cardiology, Ankara University School of Medicine, Ankara, Turkey; Department of Cardiology, Ankara University School of Medicine, Ankara, Turkey; Department of Cardiology, Ankara University School of Medicine, Ankara, Turkey; Department of Cardiology, Ankara University School of Medicine, Ankara, Turkey; Department of Cardiology, Ankara University School of Medicine, Ankara, Turkey; Department of Cardiology, Ankara University School of Medicine, Ankara, Turkey; Department of Cardiology, Ankara University School of Medicine, Ankara, Turkey

**Keywords:** acute coronary syndrome, non-ST-elevation myocardial infarction, H-FABP, CK-MB, troponin

## Abstract

**Objective:**

Heart-type fatty acid-binding protein (H-FABP) is a novel cardiac marker used in the early diagnosis of acute myocardial infarction (AMI), which shows myocyte injury. Our study aimed to compare bedside H-FABP measurements with routine creatine kinase-MB (CK-MB) and troponin I (TnI) tests for the early diagnosis of non-ST-elevation MI (NSTEMI), as well as for determining its exclusion capacity.

**Methods:**

A total of 48 patients admitted to the emergency room within the first 12 hours of onset of ischaemic-type chest pain lasting more than 30 minutes and who did not have ST-segment elevation on electrocardiography (ECG) were included in the study. Definite diagnoses of NSTEMI were made in 24 patients as a result of 24-hour follow up, and the remaining 24 patients did not develop MI.

**Results:**

When various subgroups were analysed according to admission times, H-FABP was found to be a better diagnostic marker compared to CK-MB and TnI (accuracy index 85%), with a high sensitivity (79%) and specificity (93%) for early diagnosis (≤ six hours). The respective sensitivities of bedside H-FABP and TnI tests were 89 vs 33% (*p* < 0.05) for patients presenting within three hours of onset of symptoms.

**Conclusion:**

Bedside H-FABP measurements may contribute to correct early diagnoses, as its levels are elevated soon following MI, and measurement is easy, with a rapid result.

## Objective

Acute coronary syndrome (ACS) defines the clinical conditions that develop as a result of an abrupt reduction in coronary blood flow. Unstable angina pectoris (UAP), ST-elevation acute myocardial infarction (STEMI), and non-ST-elevation acute myocardial infarction (NSTEMI) are points on this clinical spectrum. All these clinical syndromes should be rapidly diagnosed and treated.^1^,^2^

Chest pain contributes to 50% of emergency room admissions and approximately 25% of these patients are hospitalised.^3^ Patients with ACS are usually admitted with chest pain. Studies have shown that the final diagnoses of patients admitted with chest pain are acute myocardial infarction (AMI) in one-third of patients, UAP in one-third, and non-cardiac chest pain in one-third.^4^

Early diagnosis of acute chest pain is especially important and difficult in patients without persistent ST-segment elevation. Electrocardiography (ECG) is a valuable and commonly used test for the detection of ACS. The initial ECG is normal or non-diagnostic in 50% of patients with ACS.^5^ STEMI is readily diagnosed with culprit ECG findings but NSTEMI/UAP diagnoses are more challenging. Inadequate and delayed diagnoses may lead to inappropriate treatment and delays in the initiation of life-saving therapy.

Diagnostic criteria of AMI were reformed after the introduction of more sensitive and specific markers for cardiac injury (cardiac troponins, CK-MB mass) and after a better understanding of the diagnostic and prognostic importance of these markers. In the 2012 report of the European Society of Cardiology (ESC)/American College of Cardiology (ACC), the essential criterion for MI was defined as elevated cardiac markers.^6^

Heart-type fatty acid-binding protein (H-FABP) is a recently discovered cardiac biomarker. It is specific to cardiomyocytes and low-molecular weight (15 kDa) cytosolic proteins, which represent five to 15% of the cytosolic proteins of cardiac myocytes.^7^ H-FABP plays an important role in intracellular transport for β-oxidation of fatty acids in the mitochondria.^8^,^9^

H-FABP is released from the myocardium into the circulation within one to three hours of myocardial injury. Its concentration in the blood peaks within six to eight hours and decreases within 24 to 30 hours.^10^ Its properties of being abundantly available in myocardial tissue, intra-cytosolic dominancy, relative tissue specificity, and early elevation in blood and urine after AMI suggest that H-FABP may be used in the early diagnosis of ACS.^11^,^12^ Its plasma kinetics and secretion are similar to that of myoglobin, therefore, it is used as a marker for the early diagnosis of ACS.^13^

There are few studies on this topic and the results of previous studies are controversial.^14^-^19^ In our study, we aimed to evaluate the diagnostic effectiveness of H-FABP in the early diagnosis of NSTEMI and to compare it with other cardiac markers, including CK-MB and troponin I (TnI) levels.

## Methods

Forty-eight patients who were admitted to the emergency department within the first 12 hours of onset of ischaemic-type chest pain lasting for longer than 30 minutes, and who did not have ST-segment elevation on ECG, were included in the study. The patients who had newly developed left bundle branch block, who were admitted more than 12 hours after the onset of chest pain, who had chronic renal failure, chronic muscular diseases or heart failure, or who had recently experienced trauma, musculoskeletal injury or shock, were excluded from the study.

A detailed medical history was obtained from each patient and a physical examination was performed. Twelve-lead ECGs were obtained and the changes were recorded. A complete blood count, biochemical tests and urgent cardiac profiles (CK-MB mass, myoglobin and TnI levels) were obtained from venous blood. Bedside H-FABP level was also determined from the same blood sample. The patients were monitored for 24 hours, and urgent cardiac profiles and ECG monitoring were performed every six hours. NSTEMI was diagnosed in 24 patients as the result of 24-hour follow up, and the remaining 24 patients did not develop MI.

The blood samples were immediately sent to the biochemistry laboratory of our hospital to measure TnI and CK-MB levels. Blood was taken in a 5-cm^3^ plain tube and centrifuged at 3 000 rpm for 10 minutes. The serum was separated and loaded into a Beckman Coulter Access II device and analysed with chemiluminescence. Measurement of the cardiac markers in each sample was completed within 30 to 45 minutes. The reference values of the cardiac markers were < 0.04 ng/ml for TnI (< 0.04 μg/l) and 0.6–6.3 ng/ml for CK-MB (0.6–6.3 μg/l).

All patients were also tested with the CardioDetect® (Med-Rennessens, Niemcy, Poland) H-FABP immunotest. It is a rapid chromatographic immunoassay method designed for qualitative determination of H-FABP levels in blood samples. Three to four drops of capillary blood were dropped onto a CardioDetect kit and left on a flat surface for 15 minutes. Double lines were interpreted as positive, a single line was negative, and no lines was interpreted as inadequate material. H-FABP > 7 μg/l was seen as positive in this test.^20^ H-FABP was tested only once in each patient, as the number of kits was limited.

TnI and/or CK-MB elevation (verified with at least two different measurements) associated with ischaemic-type chest pain for over 30 minutes and without persistent ST-segment elevation was accepted as NSTEMI, regardless of ECG change, as recommended by the ESC/ACC committee.6

## Statistical analysis

All data were transferred to the SPSS 10.0 statistics program. The Student’s *t*-test was used for a comparison of the groups when parametric assumptions were realised, and the chi-square and Fisher’s exact tests were used as a comparison and an association of the categorical data, respectively. Screening test results are also given. A *p*-value of < 0.05 was considered statistically significant.

For calculation of sample size, as a guideline we used the results of a study conducted by Ruzgar *et al.*,^21^ in which a sensitivity of tnI and H-FABP was 0.38 and 0.95, respectively. However, in order to be more conservative, sample size was calculated based on a sensitivity of tnI of 0.38, sensitivity of H-FABP of 0.8, pre-test probability of 0.6, power of 0.8, and type 1 error rate of 0.05 (with 95% confidence). We found the required sample size to be 43, and our study group consisted of 48 people.

For an assessment of the diagnostic performance of cardiac markers in the diagnosis of NSTEMI, sensitivity, specificity, negative predictive value (NPV), positive predictive value (PPV) and the accuracy index (AI) of each marker were calculated according to admission times. Diagnostic sensitivity was calculated by dividing the number of patients who were diagnosed with NSTEMI using H-FABP, CK-MB or TnI levels by the number of patients who were diagnosed with NSTEMI, as recommended by the ESC/ACC committee.^6^ Diagnostic specificity was calculated by dividing the number of patients who were diagnosed without NSTEMI using H-FABP, CKMB or TnI levels by the number of the patients who were diagnosed without NSTEMI, as recommended by ESC/ACC committee.^6^

PPV was calculated as the ratio of the number of patients with NSTEMI with positive test results to the number of all patients with positive test results. NPV was calculated as the ratio of the number of patients without NSTEMI with negative test results to the number of all patients with negative test results. Accuracy index was the ratio of the sum of the true-positive (positive marker and NSTEMI) and true-negative (negative marker and no NSTEMI) patients to the number of all patients. The accuracy shows that a cardiac marker can be used as the criterion for an acceptable diagnostic marker for diagnosis of MI.

**Table:** 

	NSTEMI +	NSTEMI -
Test +	a	b
Test –	c	d

Sensitivity = a/(a + c)

Specificity = d/(b + d)

PPV = a/(a + b)

NPV = d/(c + d)

Accuracy = (a + d)/(a + b + c + d)

## Results

In total, 48 patients were included in the study. The demographic and clinical characteristics of the patients are given in Table 1. The mean time of admission was 5.2 (2–10) hours. While ST-segment depression was seen on the ECG of 14 patients (29%), T-wave negativity was seen in 20 (42%) and ST–T segment changes were not detected in 14 patients (29%). A diagnosis of NSTEMI was made in 24 out of 48 patients. Coronary angiography was performed in 40 patients.

**Table 1:** Patients’ characteristics

*Characteristic*	*Number (%) or mean ± SD (minimum–maximum values)*
Age	60 ± 9 (38–79)
Male gender	28 (58)
Hypertension	35 (73)
Diabetes mellitus	14 (29)
Smoking	23 (48)
Total cholesterol (mg/dl)	204 ± 57 (97–311)
(mmol/l)	5.28 ± 1.48 (2.51–8.05)
LDL-C (mg/dl)	124 ± 55 (34–243)
(mmol/l)	3.21 ± 1.42 (0.88–6.29)
HDL-C (mg/dl)	45 ± 11 (14–69)
(mmol/l)	1.17 ± 0.28 (0.36–1.79)
Triglycerides (mg/dl)	148 ± 88 (19–533)
(mmol/l)	1.67 ± 0.99 (0.21–6.02)
Family history	12 (25)
History of CAD	15 (31)
Admission time (hours)	5.2 ± 2.4 (2–10)
ECG on admission	
ST depression	14 (29)
T negativity	20 (42)
No ECG changes	14 (29)
Coronary angiography findings	
Normal coronary arteries	7 (17.5)
Insignificant stenosis (< 50%)	9 (22.5)
Single-vessel disease	13 (32.5)
Multiple-vessel disease	11 (27.5)

LDL-C, low-density lipoprotein cholesterol; HDL-C, high-density lipoprotein cholesterol; CAD, coronary artery disease.

While H-FABP assessment on admission (two to 10 hours after onset of chest pain) was positive in 20 out of 24 patients whose NSTEMI diagnoses were definite, negative results were obtained in four patients. These four patients constituted the false-negative patient group. H-FABP was found to be negative in 22 out of 24 patients in whom NSTEMI was eliminated, and it was found to be positive in two. These two patients constituted the false-positive patient group. Table 2 summarises the positivity and negativity of the cardiac markers, which were tested on admission. The results of the analyses based on these data showed that diagnostic sensitivity was 83.3%, specificity was 91.7%, NPV was 84.6%, PPV was 90.6%, and AI was 87% for H-FABP in the diagnosis of NSTEMI.

**Table 2:** Cardiac markers of the patients who were diagnosed with and without NSTEMI

	NSTEMI + (n = 24)	NSTEMI – (n = 24)
H-FABP positive, n (%)	20 (83.3)	2 (8.3)
TnI positive, n (%)	15 (62.5)	4 (16.6)
CK-MB positive, n (%)	12 (50)	1 (4.1)

Comparisons of these values with other cardiac markers are summarised in Table 3. A comparative analysis of the data obtained when the patients were divided into three groups, according to admission times (≤ three hours, three to six hours, and > six hours after onset of symptoms) is given in Table 4, and two groups (≤ six hours and > six hours after onset of symptoms) is given in Table 5. The sensitivity and specificity of H-FABP for ≤ three hours were calculated as 89 and 100%, respectively, the sensitivity and specificity for three to six hours were 70 and 89%, respectively, and the sensitivity and specificity for > six hours were 100 and 89%, respectively.

**Table 3:** Sensitivity, specificity, NPV, PPV and AI of H-FABP, TnI and CK-MB in the diagnosis of NSTEMI

	*Sensitivity (%)*	*Specificity (%)*	*NPV*	*PPV*	*AI*
H-FABP	83.3	91.7	84.6	90.9	87.5
TnI	62.5	83.3	68.9	78.9	72.9
CK-MB	50	95.8	95.8 65.7	92.3	72.9

**Table 4:** Diagnostic value of H-FABP, TnI and CK-MB in NSTEMI diagnosis, according to admission time after onset of symptoms (≤ 3, 3–6 and > 6 hours)

	*≤ 3 hours (n = 15)*	*3–6 hours (n = 19)*	*> 6 hours (n = 14)*
H-FABP			
Sensitivity (%)	89	70	100
Specificity (%)	100	89	89
NPV (%)	86	73	100
PPV (%)	100	88	83
AI (%)	93	78	92
TnI			
Sensitivity (%)	33	70	100
Specificity (%)	100	67	89
NPV (%)	50	67	100
PPV (%)	100	70	83
AI (%)	60	68	92
CK-MB mass			
Sensitivity (%)	22	50	100
Specificity (%)	100	89	100
NPV (%)	46	62	100
PPV (%)	100	83	100
AI (%)	53	68	100

**Table 5:** Diagnostic value of H-FABP, TnI and CK-MB in NSTEMI diagnosis, according to admission time after onset of symptoms (≤ 6 and > 6 hours)

	*≤ 6 hours (n = 34)*	*> 6 hours (n = 14)*
H-FABP		
Sensitivity (%)	79	100
Specificity (%)	93	89
NPV (%)	78	100
PPV (%)	94	83
AI (%)	85	93
TnI		
Sensitivity (%)	53	100
Specificity (%)	80	89
NPV (%)	57	100
PPV (%)	77	83
AI (%)	65	93
CK-MB mass		
Sensitivity (%)	37	100
Specificity (%)	93	100
NPV (%)	54	100
PPV (%)	88	100
AI (%)	62	100

The respective sensitivities of bedside H-FABP and tnI tests were 89 vs 33% (*p* < 0.05) for patients presenting within three hours of onset. When H-FABP, CK-MB and TnI were compared according to AI at ≤ three and three to six hours, H-FABP was shown to be a better diagnostic marker (*p* < 0.01 and *p* < 0.05, respectively).

From the assessment of the two groups of admission times (≤ six hours and > six hours after onset of symptoms), the diagnostic sensitivity and specificity of H-FABP levels were found to be 79 and 93% for ≤ six hours, respectively, while sensitivity and specificity were found to be 100 and 89% for > six hours, respectively. These values indicate that H-FABP is a sensitive and specific marker for the diagnosis of NSTEMI at ≤ six hours (accuracy 85%).

**Fig. 1.**
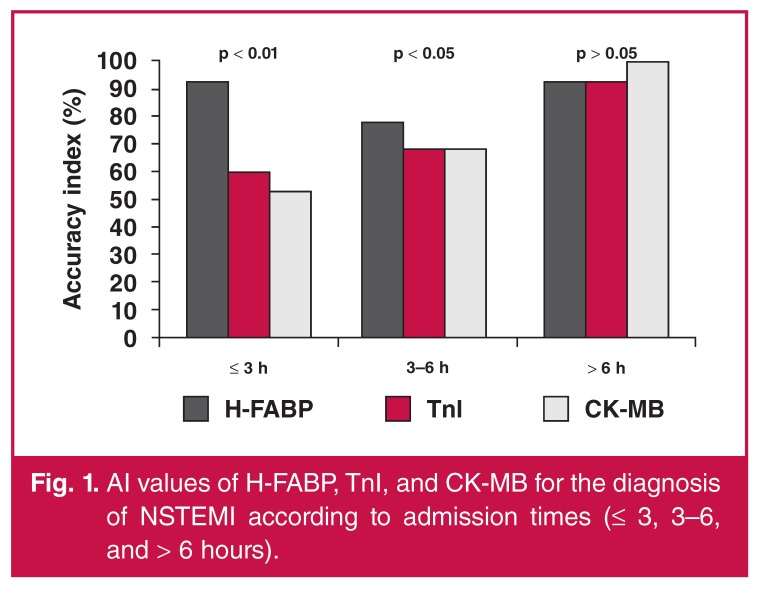
AI values of H-FABP, TnI, and CK-MB for the diagnosis of NSTEMI according to admission times (≤ 3, 3–6, and > 6 hours).

**Fig. 2.**
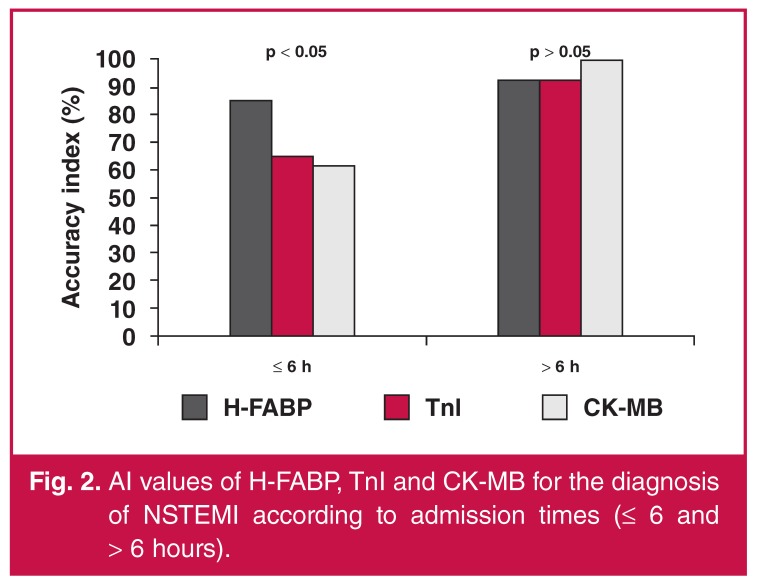
AI values of H-FABP, TnI and CK-MB for the diagnosis of NSTEMI according to admission times (≤ 6 and > 6 hours).

When AI values were compared, H-FABP was found to be a better diagnostic marker than TnI (85 vs 65%, *p* < 0.05) and CK-MB (85 vs 62%, *p* < 0.05) for the early period (≤ six hours). On the other hand, a statistically significant difference was not detected between H-FABP, TnI and CK-MB levels in the patient group that was admitted after more than six hours (*n* = 14). Fig. 1 and Fig. 2 compare the AI values of the cardiac markers according to admission times after onset of symptoms.

False-negative results were obtained in four patients who were tested for H-FABP, and false-positive results were obtained in two. Although all characteristics of these six patients were reviewed in detail with the hope of finding any predictors for false negativity and false positivity, no characteristics could be detected to explain this condition.

## Discussion

In our study, the role of bedside H-FABP measurement was investigated in 48 patients who were admitted to the emergency room within 12 hours of onset of chest pain lasting for more than 30 minutes and who did not have ST-segment elevation on ECG. It was concluded that H-FABP was a better diagnostic marker than CK-MB and Tn I, with high sensitivity (79%) and specificity (93%) (AI = 85%) for early diagnosis of NSTEMI (≤ six hours). In addition, the sensitivity and specificity of H-FABP for the group admitted ≤ three hours of onset of symptoms were calculated as 89 and 100%, respectively.

H-FABP is seen as a novel cardiac marker in the diagnosis of ACS. Nakata *et al.*^22^ and O’Donoghue *et al.*^23^ have shown it to be an early diagnostic and prognostic marker. It begins to elevate in the plasma within one to three hours following the initial symptoms of ACS and decreases to normal levels within 24 to 36 hours.^10^

A few immunohistochemical methods are used for the detection of H-FABP levels and these take from 45 minutes to 16 hours. This time decreases to 15 minutes with CardioDetect, a single-step, qualitative bedside test. Values > 7 mg/l are seen as positive.^20^ In a previous study conducted on 38 patients, this bedside method was compared with the ELISA method, which is used for the quantitative measurement of H-FABP levels, and it was completed in 45 minutes. These two methods were therefore similarly successful in making a diagnosis.^20^,^24^

Recent studies in the literature on the diagnostic value of H-FABP in ACS have given controversial results.^25^,^26^ Some studies showed H-FABP to be a reliable diagnostic tool for the early diagnosis of ACS/MI,^14^-^16^ and others displayed negative results.^17^-^19^

In the study by Glatz *et al.*^9^ conducted on 83 patients, the diagnostic sensitivity of H-FABP was shown to be better than that of myoglobin in patients who were admitted within six hours of onset of symptoms (78 vs 53%, *p* < 0.05). Similarly, in the study by Haastrup *et al.*,^27^ which was conducted on 130 patients who did not have ST-segment elevation and were admitted in under six hours, the sensitivity of H-FABP was found to be 90–95% and specificity was 81–94% for different reference values. Myoglobin and H-FABP were reported to be useful markers in the early triage of patients with chest pain.

In the study by Yoshihiko *et al.*, which was conducted on 129 patients suspected of AMI, the sensitivity of H-FABP was found to be 100% and specificity was 63% in the first three hours.^28^ Patients with STEMI were included this study. In the same study, the sensitivity of troponin T (TnT) was found to be 50% and specificity was 96% in the first three hours. They concluded that H-FABP was a more valid marker than TnT in the diagnosis of AMI.^28^ We used TnI as a marker in our study. TnI is more commonly used today and it has been found to have greater specificity for myocardial injury in chronic renal failure patients than TnT.^29^

Similarly, in the study by Ruzgar *et al.*^21^ using a qualitative H-FABP measurement, they determined the sensitivity of H-FABP as 95% in the first six hours; however, patients with ST-segment elevation were included in the study. We did not include patients with ST-segment elevation in our study because early diagnosis can be made without waiting for the results of cardiac markers in patients with chest pain accompanied by ST-segment elevation.

In a recent large study by McMahon *et al.*,^30^ H-FABP was shown to have the highest NPV of all the individual markers in the zero-to-three-hours admission time (93%) for early diagnosis of MI/ACS. According this study, H-FABP was also a valuable rule-out test for patients presenting three to six hours after the onset of chest pain. Unlike our study, H-FABP was measured quantitatively in this study.

The study by Figiel *et al.*^31^ showed similar results to ours. The difference was that we focused on the time of admission from the onset of symptoms, and determined the sensitivity, specificity, NPV and PPV, since the primary benefit of a new biomarker would be early, rapid and accurate diagnosis. H-FABP seemed to be more sensitive than TnI, with a higher NPV in patients with admission within three hours of onset of symptoms. It would be possible to rule out NSTEMI diagnosis early in the course.

In our study, diagnostic sensitivity and specificity, and the NPV and PPV of H-FABP were calculated as 83.3, 91.7, 84.6 and 90.6%, respectively, when all patients who were admitted after less than 12 hours of symptoms were evaluated. When compared with tnI and CK-MB, although the AI of H-FABP was found to be greater, the main time interval of H-FABP was superior to conventional markers at ≤ six hours. While the AI of H-FABP was 85% in this period, the AI of TnI and CK-MB were below this (65 and 62%, respectively, *p* < 0.05).

The importance of our study was that it included only patients who had long-standing ischaemic-type chest pain, it excluded patients with ST-segment elevation, and we used a qualitative bedside method of H-FABP determination (CardioDetect). The limitations of our study include the small number of patients, it was a single-centre study, the study groups consisted of only patients who had ischaemic-type chest pain, and H-FABP was tested once only in every patient.

## Conclusion

H-FABP appears to be a good diagnostic tool in the early period of NSTEMI in patients admitted with ischaemic-type chest pain. H-FABP could contribute to early bedside diagnosis in emergency rooms, as it is more sensitive and specific than other routinely used cardiac markers, such as TnI and CK-MB. This is because it becomes elevated soon after MI. Our results need to be confirmed with larger studies before routine use of this procedure.
